# Comprehensive analysis of autophagy-related gene expression profiles identified five gene biomarkers associated with immune infiltration and advanced plaques in carotid atherosclerosis

**DOI:** 10.1186/s13023-023-02660-2

**Published:** 2023-03-23

**Authors:** Chi Ma, Taoyuan Lu, Yanyan He, Dehua Guo, Lin Duan, Rufeng Jia, Dongyang Cai, Tao Gao, Zhongcan Chen, Binghua Xue, Tianxiao Li, Yingkun He

**Affiliations:** 1 Present Address: Department of Cerebrovascular Disease, Zhengzhou University People’s Hospital, Henan Provincial People’s Hospital, Zhengzhou, Henan 450003 China; 2 Henan Provincial NeuroInterventional Engineering Research Center, Henan International Joint Laboratory of Cerebrovascular Disease, and Henan Engineering Research Center of Cerebrovascular Intervention Innovation, Zhengzhou, Henan 450003 China; 3 Department of Cerebrovascular Disease, Henan University People’s Hospital, Henan Provincial People’s Hospital, Zhengzhou, Henan 450003 China; 4 Department of Neurosurgery, Zhengzhou University People’s Hospital, Henan Provincial People’s Hospital, Zhengzhou, Henan 450003 China; 5 Department of Endocrinology, Zhengzhou University People’s Hospital, Henan Provincial People’s Hospital, Zhengzhou, Henan 450003 China

**Keywords:** Carotid atherosclerosis, Autophagy-related genes, Biomarkers, Competing endogenous RNA network, Immune landscape

## Abstract

**Background:**

Autophagy plays an important role in the progression of carotid atherosclerosis (CAS). This study aimed to identify hub autophagy-related genes (ATGs) associated with CAS.

**Methods:**

GSE43292 and GSE28829 datasets of early and advanced CAS plaques were enrolled from the Gene Expression Omnibus (GEO) database. A comprehensive analysis of differentially expressed ATGs (DE-ATGs) was conducted. Functional enrichment assay was used to explore biological functions of DE-ATGs. The hub ATGs were identified by protein–protein interaction (PPI) network. Immunohistochemistry (IHC) and Real-time reverse transcription-quantitative polymerase chain reaction (RT-qPCR) were used to validate hub ATGs at the protein level and mRNA level. Correlation analysis of hub ATGs with immune cells was also conducted. In addition, a competitive endogenous RNA (ceRNA) network was constructed, and diagnostic value of hub ATGs was evaluated.

**Results:**

A total of 19 DE-ATGs were identified in early and advanced CAS plaques. Functional enrichment analysis of DE-ATGs suggested that they were closely correlated to autophagy, apoptosis, and lipid regulation. Moreover, 5 hub ATGs, including TNFSF10, ITGA6, CTSD, CCL2, and CASP1, were identified and further verified by IHC. The area under the curve (AUC) values of the 5 hub ATGs were 0.818, 0.732, 0.792, 0.814, and 0.812, respectively. Competing endogenous RNA (ceRNA) networks targeting the hub ATGs were also constructed. In addition, the 5 hub ATGs were found to be closely associated with immune cell infiltration in CAS.

**Conclusion:**

In this study, we identified 5 hub ATGs including CASP1, CCL2, CTSD, ITGA6 and TNFSF10, which could serve as candidate diagnostic biomarkers and therapeutic targets.

**Supplementary Information:**

The online version contains supplementary material available at 10.1186/s13023-023-02660-2.

## Background

Carotid atherosclerosis (CAS) is a chronic inflammatory manifestation of atherosclerosis in the carotid arteries [1]. Approximately 18–25% of ischemic strokes result from thromboembolism due to carotid atherosclerotic disease [2]. Although the pathogenesis of CAS has not been fully understood, it has been found to be involved in metabolic disorders, endothelial dysfunction, inflammation, and oxidative stress [3]. Autophagy plays an important role in the progression of CAS, while the underlying mechanism has still remained elusive [4].

Autophagy, an intracellular conserved self-degradation system via the lysosomal pathway, is a crucial core molecular pathway for maintaining cellular and organismal homeostasis. Dysregulation of autophagy is closely related to the pathogenesis of many human diseases [5, 6]. Autophagy plays an important role in a variety of pathologies, such as immunity, cell death, cardiovascular and cerebrovascular diseases, and kidney and liver diseases, which may be mediated by evolutionarily conserved autophagy-related genes (ATGs) [7].

Autophagy has been reported to play cytoprotective functions, and it represents an essential in vivo process, mediating vascular functions. In the formation of CAS, autophagy is activated in all major cell types, such as vascular smooth muscle cells (VSMCs) and macrophages [8, 9]. However, it has been shown that the abnormal expression of some ATGs were closely related to the development of advanced CAS plaques. For example, abnormal expression of vital ATGs (Atg5, Atg7) in macrophages and VSMCs may accelerate the formation of atherosclerotic plaques in mice [10–13]. In addition, MAP1LC3B, a gene mainly associated with autophagy, had also been shown to be associated with inflammation, which may require the removal of dead cells from atherosclerotic lesion sites. It was also found that low expression of MAP1LC3B did not benefit from MAP1LC3B-associated autophagy [14].

CAS is a major and potentially preventable cause of ischemic stroke. Carotid endarterectomy and stenting are proven techniques for primary prevention of stroke, although the number of procedures required to prevent a single stroke in asymptomatic patients remains noticeable [15]. However, with the advancement of technology, brain protection and careful patient selection, drug-eluting stents [16], nanocarriers [17] and exosome poly-ubiquitin therapy [18] are becoming alternative technologies for stroke prevention and have broad therapeutic prospects for CAS. Thus, we attempted to find out some new therapeutic targets. Transcranial Doppler for microemboli, double- ultrasound for plaque echogenicity, and brain computed tomography/magnetic resonance imaging (CT/MRI) for asymptomatic embolic infarcts are all routine imaging modalities for CAS [19]. The high cost of imaging studies and the difficulty of early intervention of CAS, however, hinder the promotion of clinical translation. In summary, regarding the important role of autophagy in CAS, as well as current limited research on CAS-related autophagic markers, exploring new autophagic markers is of great significance for early diagnosis and treatment of CAS.

In the present study, we attempted to explore differentially expressed ATGs (DE-ATGs) of early and advanced CAS plaques, and to identify the hub ATGs in the progression of CAS using a variety of bioinformatics methods. The correlation of immune cells and the diagnostic value of the hub ATGs was also assessed. In addition, potential competitive endogenous RNA (ceRNA) regulatory networks were constructed, which could provide a reference to deeply understand the molecular mechanism and therapeutic targets of CAS.

## Results

### Identification of DE-ATGs

**Fig. 1:**
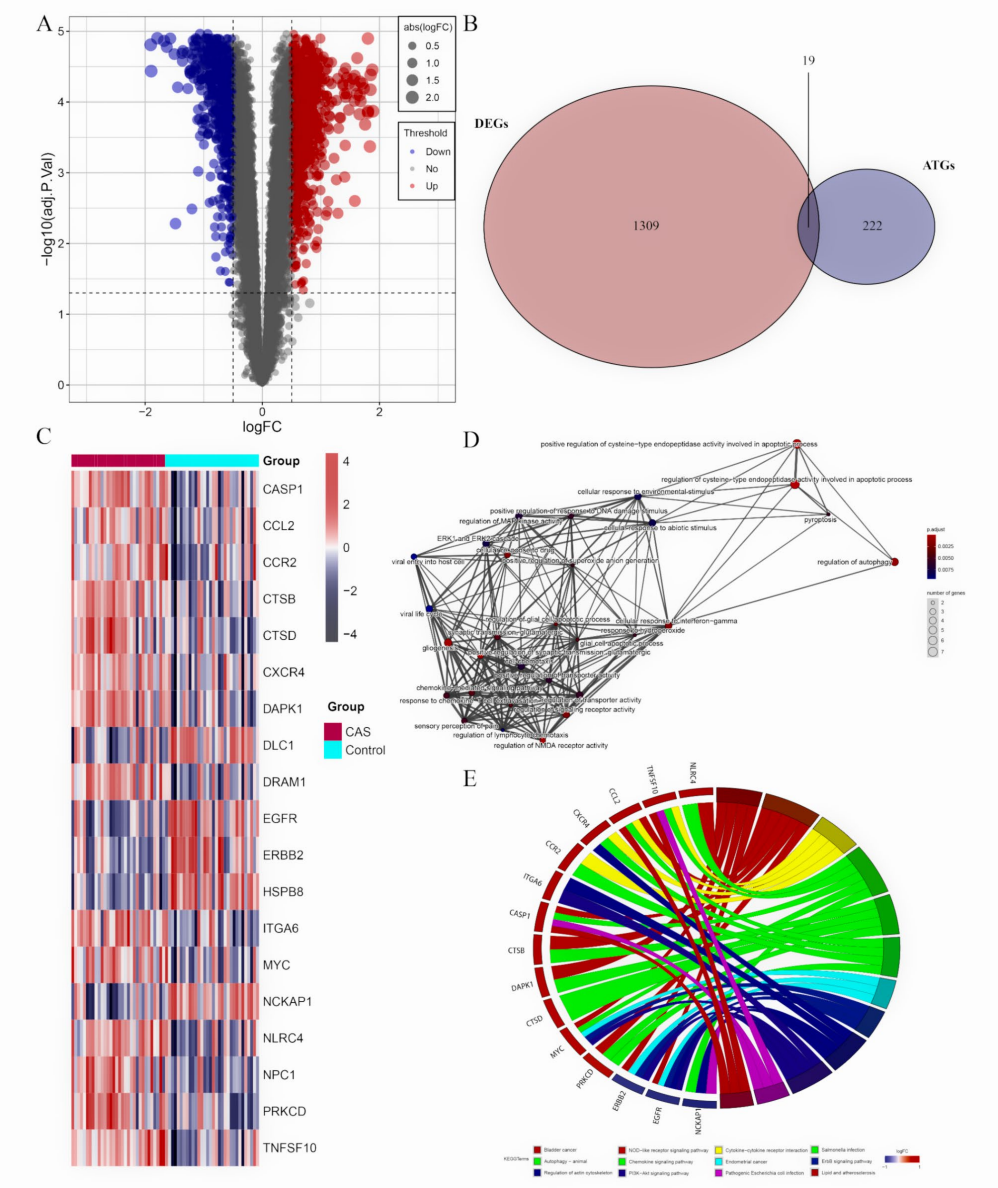
**The differentially expressed autophagy-related genes (DE-ATGs) for CAS and control samples.** (**A**) A volcano plot for early and advanced CAS plaques. (**B**) The Venn plot of the DE-ATGs. (**C**) The heatmap of the DE-ATGs. (**D**) The GO enrichment and (**E**) KEGG pathway enrichment of DE-ATGs

The details of the two datasets are presented in Table 1. The matrix file from GSE43292 was pre-processed and annotated with the official gene symbol. A total of 1328 DEGs were identified in the GSE43292, of which 594 were down-regulated and 734 were up-regulated (Fig. 1A). In addition, 19 DE-ATGs were obtained by taking intersections of the DEGs with the 222 ATGs (Fig. 1B). The heatmap of the 14 up and 5 down-regulated DE-ATGs is shown in Fig. 1C.

**Table 1:** Baseline information for two data sets in this study

Parameters	GSE43292	GSE28829
**Platform**	GPL6244 Affymetrix Human Gene 1.0 ST Array	GPL570 Affymetrix Human Genome U133 Plus 2.0 Array
**Type**	Microarray	Microarray
**N**	64	29
**Group (%)**		
CAS	32(50)	16(55)
Control	32(50)	13(45)

### GO and KEGG enrichment analyses

The GO and KEGG pathway enrichment analyses were conducted to explore the potential biological functions of the DE-ATGs. The results of the GO analysis suggested that the DE-ATGs were significantly associated with apoptosis-related biological processes, such as regulation of autophagy, positive regulation of cysteine − type endopeptidase activity involved in apoptosis process, regulation of signaling receptor activity, regulation of glial cell apoptosis process, and positive regulation of response to DNA damage stimulus (Fig. 1D). The results of the KEGG pathway enrichment analysis indicated that the DE-ATGs were mainly enriched in autophagy, lipid, and carotid atherosclerosis, PI3K − Akt signaling pathway, NOD-like receptor signaling pathway, and cytokine-cytokine receptor interaction, indicating the autophagy and immune activation, as well as lipid metabolism disorders (Fig. 1E). The DE-ATGs were closely correlated to autophagy and immunity in CAS.

### Construction of the PPI network and screening of hub ATGs

**Fig. 2:**
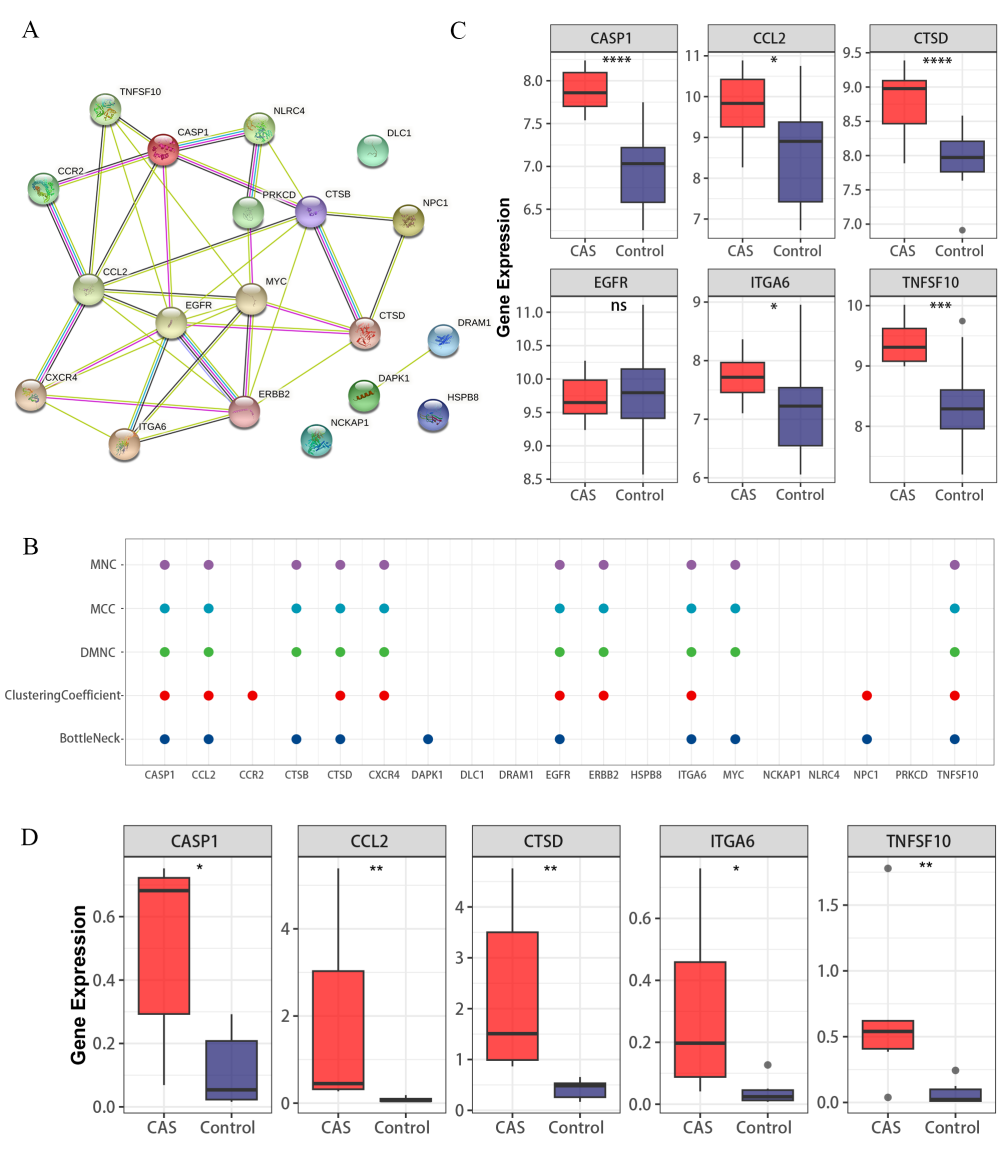
**Identification of key ATGs.** (**A**) The PPI networks. (**B**) Scatter plot. Scatter plot of the overlapping ATGs for the top 10 ATGs selected based on the 5 ranking methods (MNC, MCC, DMNC, ClusteringCoefficient, and BottleNeck). (**C**) 6 hub ATGs were validated in the GSE28829 dataset. (**D**) Validation of 5 key genes at the mRNA level. ns > 0.05; *P < 0.05; **P < 0.01; ***P < 0.001; ****P < 0.0001

To explore the functional connectivity of the DE-ATGs, we interrogated the STRING database to construct a PPI network (Fig. 2A). The “cytoHubba” plugin of the Cytoscape software was used to identify hub ATGs. The top 10 hub genes were recognized using MNC, MCC, DMNC, ClusteringCoefficient, and BottleNeck algorithms, of which 6 hub ATGs were retained after taking the intersection (Fig. 2B). Furthermore, the GSE28829 dataset was employed to validate the expression levels of these 6 hub ATGs, and found no significant difference in the expression of epidermal growth factor receptor (EGFR) (Fig. 2C). Eventually, 5 hub ATGs, including CASP1, CCL2, CTSD, ITGA6, and TNFSF10 were considered as hub ATGs, which were involved in the pathological progression of CAS.

### Validation of hub ATGs at the mRNA and protein level

**Fig. 3:**
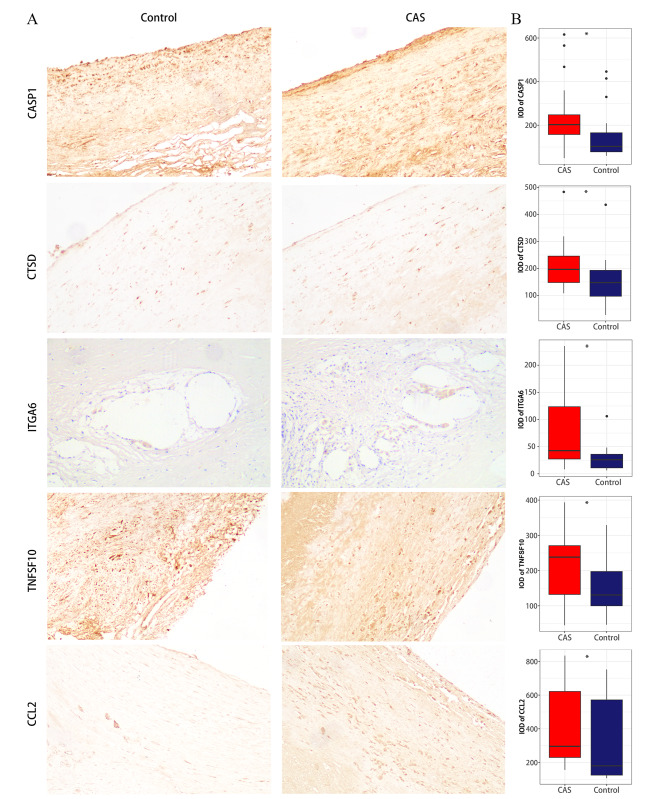
**The results of IHC.** (**A**) IHC images of the 5 hub ATGs in CAS and control samples under a 200X microscope. (**B**) The IOD of the 5 hub ATGs. ns > 0.05; *P < 0.05; **P < 0.01; ***P < 0.001; ****P < 0.0001

Our RT-qPCR results also support the above analysis results (Fig. 2D). As shown in Fig. 3A, the protein expression levels of the 5 hub ATGs were relatively higher in advanced CAS plaques. Further quantitative analysis indicated that the expression levels of CASP1 (P = 0.024), CTSD (P = 0.031), ITGA6 (P = 0.016), TNFSF10(P = 0.027) and CCL2 (P = 0.029) genes in early and advanced plaques were significantly different (Fig. 3B).

### Diagnostic value of hub ATGs

**Fig. 4:**
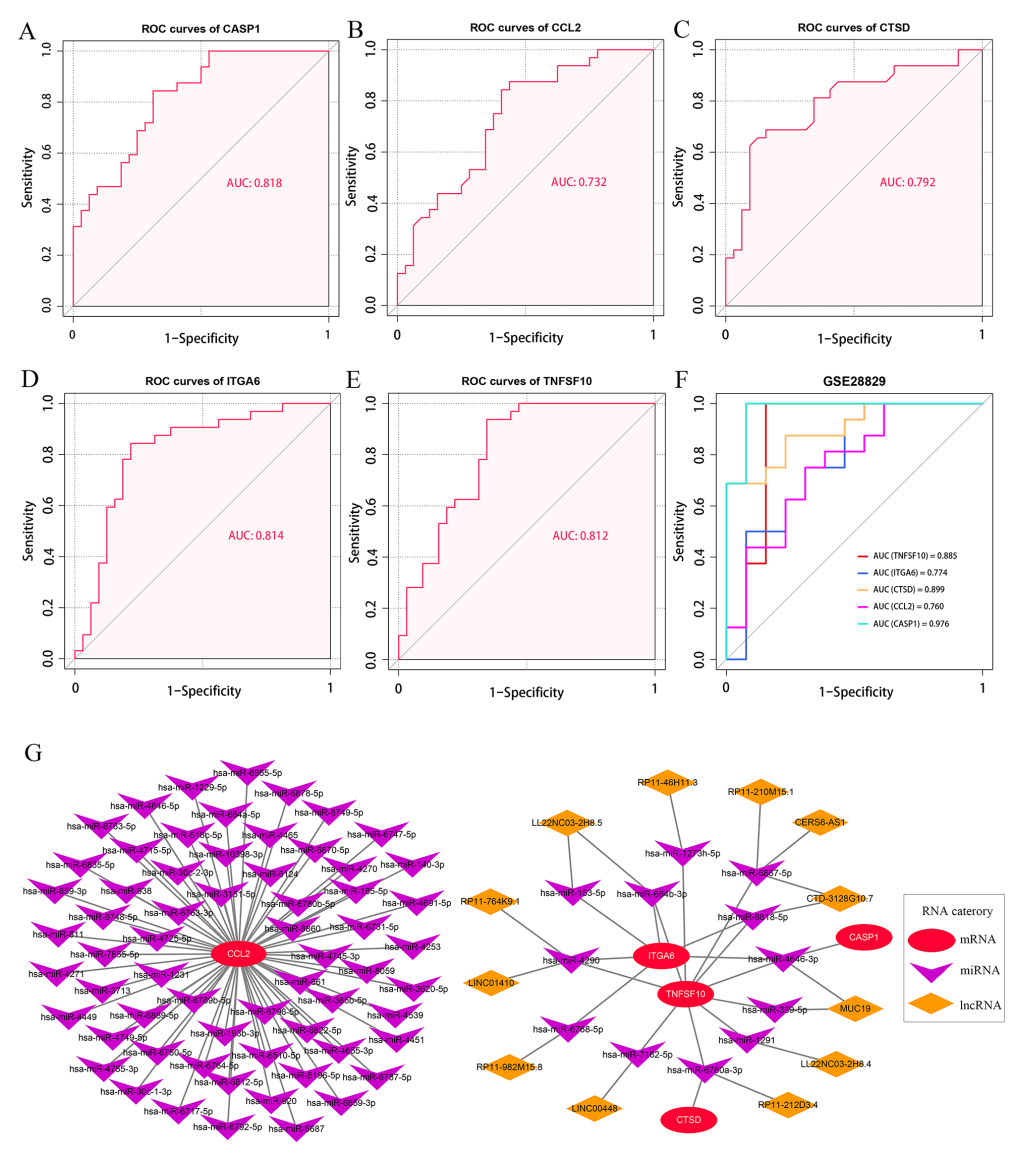
**ROC curves of the five hub ATGs in the diagnosis of CAS and ceRNA network.** (**A-E**) ROC curves of the 5 hub ATGs in the GSE43292 dataset. (**F**) ROC curves of the 5 hub ATGs in the GSE28829 dataset. Different colored curves represent the different hub ATGs. (**G**) The mRNA-miRNA-lncRNA network

The results of the ROC curve analysis demonstrated that all the 5 hub ATGs possessed a good diagnostic value for advanced CAS. The ROC curves of CASP1, CCL2, CTSD, ITGA6, and TNFSF10 were visualized in Fig. 4A-E. The values of area under the curves (AUC) of CASP1, CCL2, CTSD, ITGA6, and TNFSF10 was 0.818, 0.732, 0.792, 0.814, and 0.812, respectively. Moreover, an external validation of the diagnostic value of the hub ATGs in the GSE28829 dataset was performed. The AUC of CASP1, CCL2, CTSD, ITGA6, and TNFSF10 in the GSE28829 dataset was 0.885, 0.774, 0.899, 0.760, and 0.976, respectively (Fig. 4F), which indicated the good stability of hub ATGs as diagnostic markers for advanced CAS plaques. In addition, we performed ROC curve analysis in GSE43292 for the logistic regression model established by combining the 5 key ATGs, while ROC curve analysis was performed in the GSE28829 test set. AUC values from the logistic regression model were 0.864 and 0.976, respectively. Detailed figures information is shown in Additional file 1: Figure S1 and Figure S2.

### Potential ceRNA regulatory network of the hub ATGs

MicroRNAs (miRNAs) are well known to induce gene silencing and to down-regulate gene expression by binding to mRNAs. In contrast, lncRNAs can endogenously compete for binding to miRNAs and consequently up-regulate the expressions of mRNAs. A ceRNA network consisting of 4 hub ATGs, 12 miRNAs and 12 lncRNA, and a miRNA-mRNA regulatory network consisting of CCL2 and 63 miRNAs were established according to the ceRNA network construction process (Fig. 4G). In these networks, lncRNAs could promote the expression levels of hub ATGs by inhibiting the corresponding miRNAs. For instance, LINC00448 could inhibit has-miR-7162-5p, and thus, up-regulate the expression level of TNFSF10. Notably, RP11-212D3.4 might be able to affect both TNFSF10 and CTSD through a has-miR6780a-3p. Additionally, TNFSF10 was simultaneously regulated by 11 IncRNA regulators, including LINC01410, RP11-764K9.1, LL22NC03-2H8.5, RP11-46H11.3, RP11-210M15.1, CERS6-AS1, CTD-3128G10.7, MUC19, LL22NC03-2H8.4, RP11-212D3.4, and LINC0048, which could be potential RNA agents or target candidates for anti-inflammatory and molecular therapy.

### Immune cell infiltration analysis

**Fig. 5:**
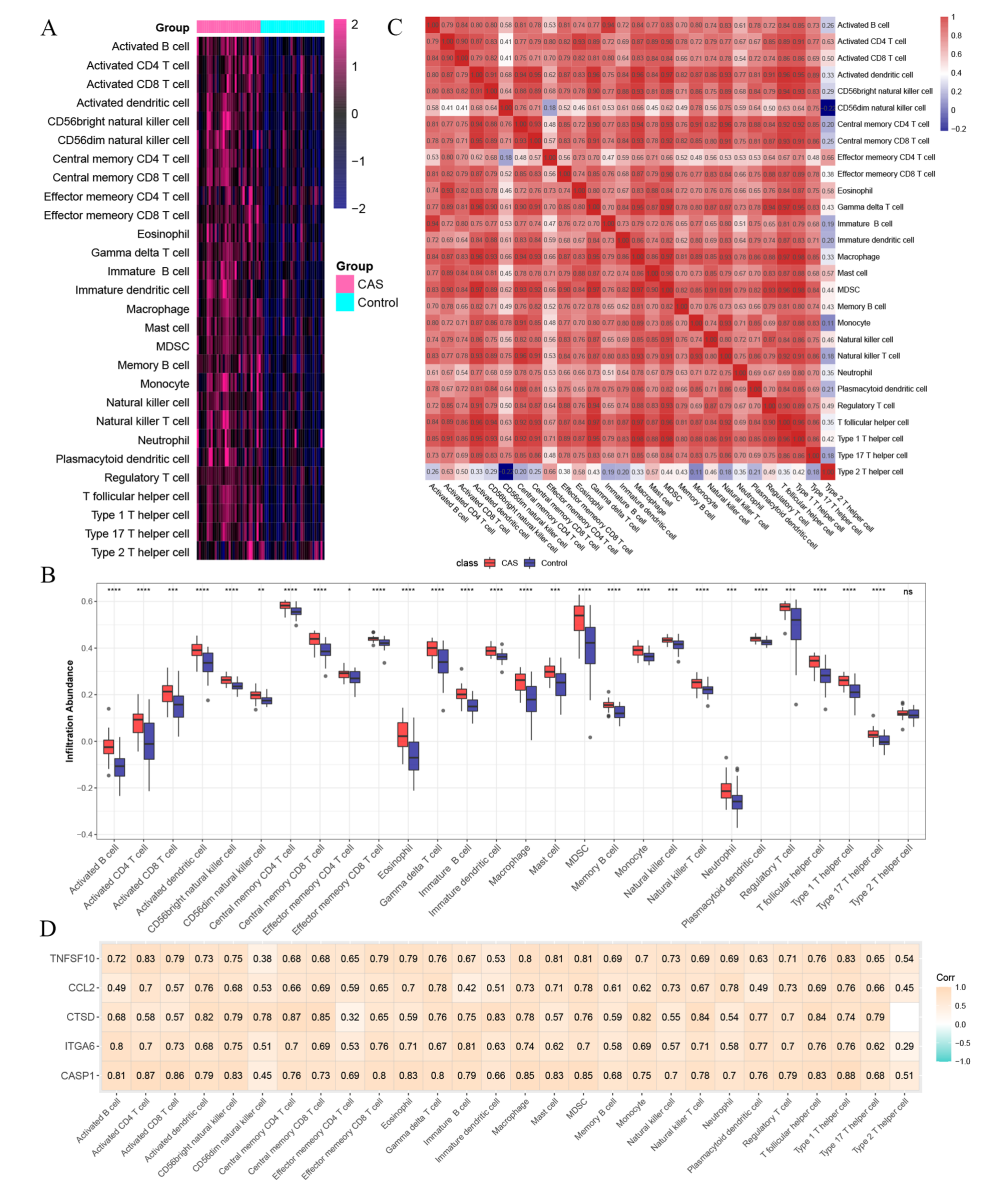
**Immune cell infiltration analysis.** (**A**) Heatmap of the proportions of 28 immune cells in CAS and control groups. (**B**) A box plot of the immune cell proportions in CAS and control groups. The control group is marked in blue, and the CAS group is marked in red. (**C**) A correlation matrix of immune cell proportions in CAS and control groups. (**D**) Correlation analysis of hub ATGs and immune cells. ns > 0.05; *P < 0.05; **P < 0.01; ***P < 0.001; ****P < 0.0001

The immune landscape of CAS plaques is illustrated in Fig. 5A-B. Compared with early CAS plaques, advanced CAS plaques exhibited a higher infiltration abundance of most immune cells, such as macrophages, activated CD4 T cells, activated CD8 T cells, myeloid-derived suppressor cells (MDSCs), neutrophils, natural killer (NK) cells, etc. Besides, we further explored the correlation between 28 immune cells, and it was revealed that most immune cells were closely correlated with each other, while only type 2 T helper cells were negatively correlated with CD56^dim^ NK cells (Fig. 5C). At the same time, in the validation dataset, it was found that there were significant differences in the expression of immune cells except CD56dim natural killer cell, Eosinophil, Neutrophil, Plasmacytoid dendritic cell, and Type 2 T helper cell. Detailed figures information is shown in Additional file 1: Figure S3. These results showed that most of the immune cells were activated in CAS. It was found that CASP1, CCL2, CTSD, ITGA6, and TNFSF10 were positively correlated with immune cells (Fig. 5D). Based on RNA-seq data from GSE43292, we calculated the proportion of 22 immune cell types in early and advanced plaques separately using CIBERSORT. Detailed figures information is shown in Additional file 1: Figure S4A-B. Alternatively, we compared the proportion of early and advanced plaque immune cell infiltration. Detailed figures information is shown in Additional file 1: Figure S4C. Advanced plaques had higher levels of Macrophages M0, Macrophages M2, Neutrophils, and T cells CD4 memory activated than early plaque, while early plaque had higher levels of B cells naïve, Monocytes, NK cells activated, Plasma cells, T cells CD8, and T cells regulatory (Tregs) than advanced plaque. Collectively, the activation of autophagy in the progression of CAS could be accompanied by the enhanced inflammation at the lesion site.

## Discussion

CAS is characterized by inflammation and lipid accumulation, and advanced plaques are more unstable and ruptured than early plaques [20]. Autophagy is an important cellular survival mechanism for cellular homeostasis that has emerged in recent years, and it may play a protective role in CAS [4]. Hence, understanding the molecular mechanism of autophagy in the development of CAS is crucial for the molecular diagnosis and treatment of CAS.

Considering the important role of autophagy in CAS, we explored the clinical significance of hub ATGs in CAS. Two relatively large sample datasets from the GEO database were utilized in this study to identify 19 DE-ATGs in early and advanced CAS plaques. GO and KEGG functional analysis of the DE-ATGs showed that cell death and metabolic disorder-related pathways, such as the regulation of autophagy, lipid, and carotid atherosclerosis, and cell apoptosis, were significantly enriched. Subsequently, 5 hub ATGs were identified from the PPI network consisting of these DE-ATGs. IHC and RT-qPCR also confirmed the difference of hub ATGs expression between early and advanced plaques. The ROC curve analysis suggested that 5 hub ATGs had a good diagnostic value for advanced CAS. Additionally, we also detected that the hub ATGs were positively correlated with a variety of immune cells, implying their latent interaction in CAS. CeRNA networks were further constructed to present the potential post-transcriptional regulatory mechanisms of the hub ATGs, which provided a target reference for molecular therapy. Overall, 5 hub ATGs identified in this study may serve as diagnostic biomarkers and therapeutic targets in CAS, contributing to advances in precision medicine.

We explored the function of 5 hub ATGs in more depth. Several studies have shown that CASP1 and CCL2 play a crucial role in promoting inflammatory responses and autophagy-related defects. CASP1 is one of the constituent components of inflammatory bodies and is activated through the formation of inflammasome complexes in response to both pathogen-derived and endogenous mediators. In immune cells, active CASP1 activates the inflammatory cytokines interleukin-1β (IL-1β) and IL-18 into a form in which they respond to inflammatory stimuli [21]. Interaction of autophagy with NLR family pyrin domain containing 3 (NLRP3) inflammasome, some activators of inflammasome such as intracellular endogenous activators (DAMPs) were found to lead to the activation of inflammasome, further leading to the activation of CASP1 and the release of proinflammatory factors. In diseases with NLRP3 inflammasome, autophagy plays a protective role in the body by inhibiting the NLRP3-CASP1 pathway through the lysosomal pathway [22]. Autophagic dysfunction could lead to diseases with excessive NLRP3 inflammasome activation and excessive inflammation. Thus becoming a major regulator of the inflammasome. In a mouse model of CAS, CASP1 promoted CAS by enhancing the inflammatory status of the lesion through a mechanism of involving activation of lesion-associated immune cells and interferon (IFN)-γ expression [23]. Monocyte chemoattractant protein-1 (MCP-1/CCL2) belongs to the chemokine family and is one of the key chemokines that regulate monocyte/macrophage migration and infiltration. Expression of CCL2 and its receptor CCR2 has been shown to be necessary for immune monitoring in inflammatory responses [24]. CCL2 promotes inflammation, induces the release of other inflammatory mediators to form a positive feedback effect, and eventually accelerates CAS progression [25]. CCL2 expression via enhanced cellular senescence induced by autophagic failure and the exposure of atherogenic lipids appeared to trigger the accumulation of monocytes and macrophages in the vascular wall [12]. However, some valuable results were obtained. Therefore, it was found that the increase in CASP1 and CCL2 could be due to the combination of autophagy-related defects and inflammation. In summary, inflammation could regulate the autophagic process, and autophagy could also regulate the inflammatory response. Activation of autophagy could balance the required host defense inflammatory response and prevent excessive and harmful inflammatory responses.

CTSD (cathepsin D), an aspartic protease, is a lysosomal enzyme that is present in autolysosomes during autophagy [26, 27]. CTSD promotes maturation through autophagy leading to cell apoptosis [28]. Autophagy was activated in CAS and is important for the maturation of CTSD. The expression levels of members of the cathepsin family (CTSD/CTSB) were up-regulated in patients with coronary heart disease [29]. Therefore, we could infer that increased CTSD is a protective factor for patients with CAS. We could also explore maintaining CTSD levels through pharmacological interventions to further enhance lysosomal function, protect the brain from stroke-induced cell death, and improve patient outcomes.

TNFSF10 encodes a protein that is a cytokine belonging to the tumor necrosis factor (TNF) ligand family. TNFSF10 has been reported to induce autophagy in some cancer cells, including prostate cancer, lung cancer, bladder cancer and other cancer cells [30]. MAPK8 was found to be a critical pathway for TNFSF10-induced autophagy by knockdown of TRAF2 and RIPK1. TNFSF10-induced autophagy was found to be cytoprotective by inhibiting MAPK8 which could improve the sensitivity of tumor cells to targeted drugs [31]. In the present study, we concluded that TNFSF10-induced autophagy has a protective effect on cells. TNFSF10 had been reported to be expressed in atherosclerotic plaques, while TNFSF10 protein expression was significantly reduced in a control artery representing non-diseased vessels (the left internal mammary artery). [32]. For patients with CAS, TNFSF10 activated autophagy to inhibit apoptosis and further delays the progression of CAS.

The extracellular matrix (ECM) interacts with cell adhesion molecules (CAMs), mainly a family of integrins and glycoproteins. These interactions lead to a range of physiological and pathological processes, such as inflammation, thrombosis, cell apoptosis, cell proliferation, and cell adhesion [33]. ITGA6, a member of the integrin family, plays a key role in the interaction between several cell types and participates in multiple biological processes, including cell proliferation and invasion [34]. Among the ATGs in female lung adenocarcinoma, there was a significant relationship between ITGA6 and its survival prognosis [35]. The present study showed that the autophagy-related gene, ITGA6, significantly increased in the progression of CAS. However, the underlying molecular mechanism of how ITGA6 can affect the progression of CAS through autophagy is still unclear and needs to be further studied.

The above studies further explored the potential clinical application value of 5 hub ATGs. In recent years, CAS has been recognized as an important cause of ischemic stroke, particularly thromboembolism due to advanced plaques [2]. Advanced plaques therefore require prompt screening diagnosis and clinical intervention to reduce the risk of stroke onset. We explored the AUC of advanced plaques and found that 5 hub ATGs had good diagnostic value and stability. Additionally, we built a logistic regression model combining the 5 key genes, with higher AUC values in both the training and test sets, with better diagnostic value and stability. In summary, 5 key autophagy genes could be used as potential biomarkers for the diagnosis of early and advanced plaques.

CAS is mediated by both inflammation and cellular immunity. Single-cell sequencing of human CAS plaques showed that most of the immune landscape of CAS plaques was macrophages and T cells [36]. However, the contribution of other immune cells has still remained elusive. We assessed the abundance of immune cell infiltration (ssGSEA) and the proportion of infiltration (CIBERSORT) in early and advanced plaques. In the current study, we found that the vast majority of immune cells were significantly increased in advanced plaques, except for type 2 T helper cells. In addition, the above immune infiltration results were verified by GSE28829 dataset, and it was found that the immune cell changes in early and advanced CAS plaques were basically consistent. From the results of CIBERSORT analysis, M0 macrophages and M2 macrophages were significantly increased in the late plate. Previous studies have found that macrophages were involved in the development of atherosclerosis when stimulated by oxidized lipids, cholesterol crystals, and inflammatory cytokines [37], which also argued for our CIBERSORT analysis results. In CAS, massive cholesterol crystals accumulate in blood vessels leading to vascular inflammation promoting the differentiation of adaptive T cells into effector and memory T cells [38]. As previous studies have shown, as CAS progresses, CD8 T cells significantly increase and plaques become prone to rupture, but they show a decrease in fibrotic calcified plaques and healing of plaque rupture [39]. Compared with early plaques, advanced plaques had increased infiltration scores, which indicated that the abundance of immune cells and the proportion of immune cells also changed. Because of significant changes in immune cells in early and advanced CAS, we reasoned that changes in immune cells could be used to describe differences between early and advanced individuals. Meanwhile, 5 hub ATGs were positively correlated with immune inflammatory cells. We also suggested that the enhancement of plaque inflammation is the result of a combination of hub ATGs with multiple immune cells in advanced CAS. Therefore, these 5 hub ATGs could be used as potential therapeutic targets for the early clinical intervention and to delay CAS progression and to stabilize plaques.

In recent years, targeted drugs and interventional therapy have developed rapidly in the context of precision medicine. Within 80% of patients undergoing carotid endarterectomy are unnecessarily exposed to surgical risks [40, 41]. We will develop more targeted drugs and interventional therapies based on these hub ATGs to reduce the risk of unnecessary surgery. Functional analysis of 5 hub ATGs revealed that the increased expression of CASP1 and CCL2 was associated with autophagy-related defects and inflammatory activation, while the increased expression of CTSD, ITGA6 and TNFSF10 was associated with autophagy-related activation. Activation of autophagy led to apoptosis, and the fragments of apoptosis further aggravate inflammation. This suggests that we should treat CAS with anti-inflammatory therapy, while activating autophagy to delay and develop the disease. In the ceRNA network, we can also activate the hub ATGs for targeted therapy of CAS through the inhibitory effects of lncRNAs on miRNAs.

There are some limitations in the present study. On the one hand, because only two datasets were included, the sample size was not very large; on the other hand, the role of 5 hub ATGs in CAS and regulatory relationships in ceRNA networks required more experimental validation. Finally, for changes in immune cells in early and late plaques, we only employed bioinformatics analysis, which needs further validation.

## Conclusions

In the present study, we identified 5 hub ATGs (CASP1, CCL2, CTSD, ITGA6, and TNFSF10) associated with progression of CAS and immune infiltration. A ceRNA network was constructed to provide reference for mechanism understanding and molecular therapy. These 5 hub ATGs also have good diagnostic value.

## Materials and methods

### Data acquisition and preprocessing

**Fig. 6:**
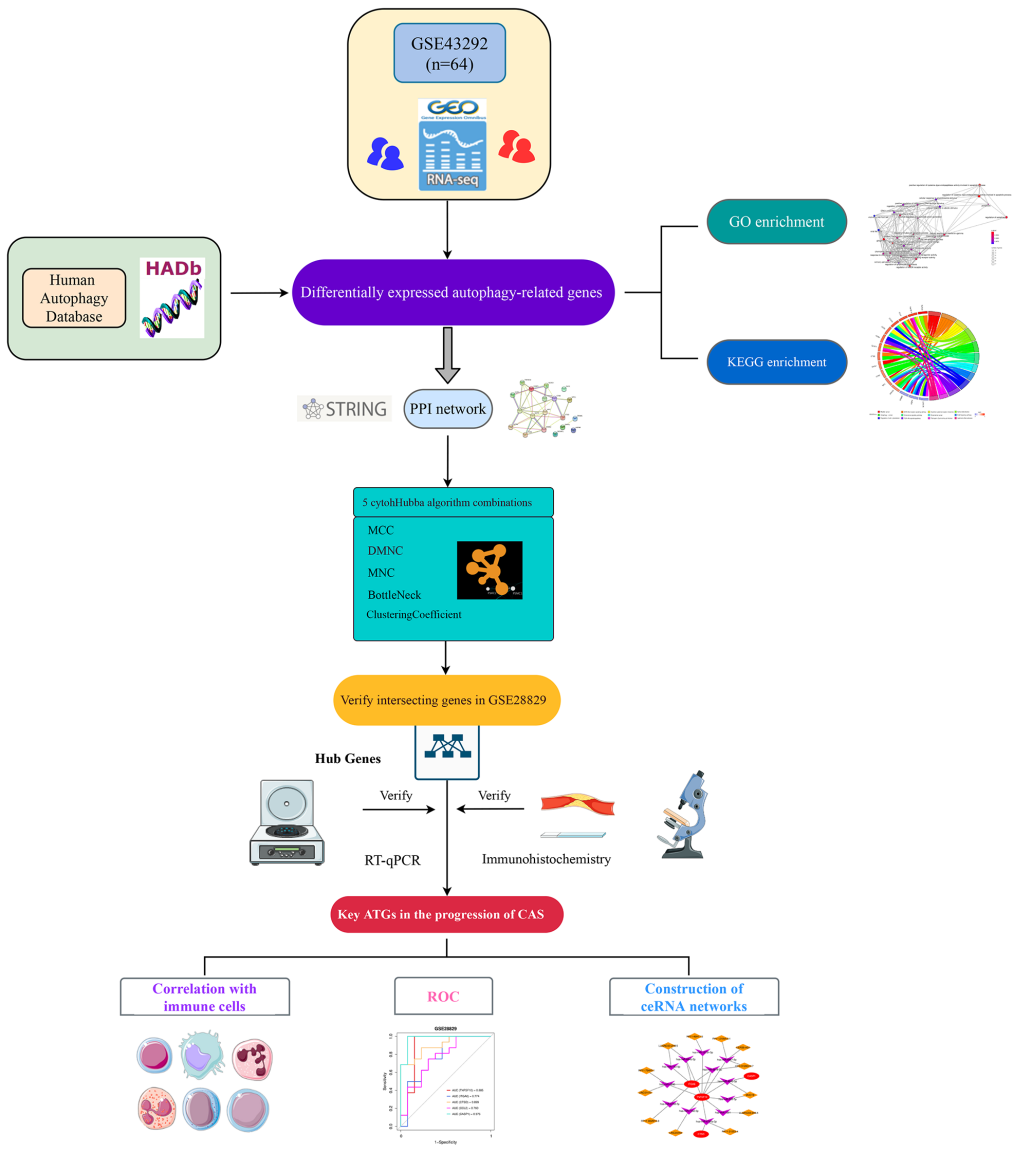
Workflow of the whole study

The workflow of this study is shown in Fig. 6. Two CAS gene expression profiles (GSE43292 and GSE28829) were acquired from the Gene Expression Omnibus (GEO) database. The GSE43292 dataset consists of 32 early CAS plaques and 32 advanced CAS plaques. The GSE28829 dataset based on the GPL570 platform consists of 13 early CAS plaques and 16 advanced CAS plaques. Human autophagy database provided an entire set of 222 human genes associated with autophagy (http://www.autophagy.lu/). Detailed database information is shown in Additional file 2: Table S1. The microarray data were preprocessed via quantile normalization and log2 transformation via the “*limma*” R package. The GENCODE database (https://www.gencodegenes.org/) was applied for mRNA and long non-coding RNA (lncRNA) annotations.

### Identification of differentially expressed autophagy-related genes (DE-ATGs)

The differentially expressed genes (DEGs) were identified between early and advanced atherosclerotic plaques by using “*limma*” R package. The genes with adjusted P-value (adj.P.Val) < 0.05 and |log-FC| > 0.5 were DEGs. DEGs were intersected with the 222 ATGs to obtain DE-ATGs.

### Enrichment analysis of DE-ATGs

To explore the biological changes associated with DE-ATGs,the Gene Ontology (GO) and Kyoto Encyclopedia of Genes and Genomes (KEGG) enrichment analyses were performed using the “*clusterProfiler*” R package. GO terms and KEGG pathways with adjusted P-value < 0.05 were considered as significantly enriched.

### Construction of protein–protein interaction (PPI) network and identification of hub genes

We constructed a PPI network using the Search Tool for the Retrieval of interacting Genes (STRING, https://cn.string-db.org/), in order to obtain insights into protein interactions between DE-ATGs. The PPI network was visualized using the Cytoscape 3.8.2 software. Then, the cytoHubba plugin was employed to extract hub ATGs from the PPI network. Top 10 hub genes were identified using MNC, MCC, DMNC, ClusteringCoefficient, and BottleNeck algorithms, and the intersection genes were deemed as hub ATGs. Furthermore, the expression levels of these hub ATGs were validated in the GSE28829 dataset.

### RNA extraction and real-time reverse transcription-quantitative polymerase chain reaction (RT-qPCR)

Six early plaque and advanced plaque tissues were obtained from patients who underwent carotid endarterectomy in the Department of Neurosurgery, People’s Hospital of Zhengzhou University. All patients > 18 years of age who provided written informed consent prior to enrollment. Total RNA was isolated from early and advanced plaques using Trizol (Thermo Fisher Scientific, USA) according to the manufacturer’s instructions. The quality and quantity of RNA were detected by absorbance at 260/280 nm using a NanoDrop One spectrophotometer (Thermo Fisher Scientific, USA). After reverse transcription of cDNA, qPCR reactions were performed with StepOne™ software (Thermo Fisher Scientific). All mRNA expression was calculated using the 2^−ΔΔCt^ method. Detailed sequences information is shown in Additional file 3: Table S2.

### Human specimen histology and immunohistochemistry (IHC)

This study was approved by the Ethics Committee of People’s Hospital of Zhengzhou University (Zhengzhou, China). Six early tunica lutea and advanced plaque tissues were obtained from patients who underwent carotid endarterectomy in the Department of Neurosurgery, People’s Hospital of Zhengzhou University. All patients aged > 18 years old and gave written informed consent prior to enrollment. After removing paraffin, hydration, and sealing, the specimens were incubated with anti-CASP1 (1:200; 22915-1-AP; Proteintech, Rosemont, IL, USA), anti-CCL2 (1:200; 66272-1-Ig; Proteintech), anti-CTSD (1:600; ab75852; Abcam, Cambridge, UK), anti-ITGA6 (1:500; ab181551; Abcam), and anti-TNFSF10 (1:50; ab231063; Abcam) antibodies overnight at 4 °C. Besides, 3,3-diaminobenzidine (DAB; Solarbio, Beijing, China) was used for chromogenic reactions. Additionally, the results of IHC were quantified using the integrated optical density (IOD) via Image-Pro Plus 6.0 software.

### Diagnostic value of hub ATGs

Receiver operating characteristic (ROC) curve analysis was employed to investigate the diagnostic value of these hub ATGs for discriminating advanced plaques from early plaques via “*pROC*” R package in GSE43292. Meanwhile, the GSE28829 dataset was used as an external validation cohort to test the diagnostic value and stability of the hub ATGs. In addition, we used GSE43292 as the training set, combined these key genes to build a logistic regression model, and finally used GSE28829 as the test set to test the model.

### Construction of CeRNA networks

The ceRNA regulatory network of model genes was constructed through the following pipelines: Firstly, miRNAs targeting 5 hub ATGs were predicted via miRWalk (http://mirwalk.umm.uni-heidelberg.de/), and filtered with miRWalk score = 1. Secondly, target lncRNAs of these miRNAs were predicted according to lncBase v.2 with a threshold of miTG score = 1. Thirdly, the miRNA-mRNA and lncRNA-miRNA binding pairs obtained from the above-mentioned steps were merged into multiple lncRNA-miRNA-mRNA regulatory axes. In addition, the ceRNA network was visualized using the Cytoscape software.

### Evaluation of immune cell infiltration in CAS

Single-sample gene-set enrichment analysis (ssGSEA) implemented in the *GSVA* R package was conducted to estimate the relative abundance of 28 immune cells in each sample, according to the marker gene set proposed by Charoentong et al. [42]. Detailed gene sets information is shown in Additional file 4: Table S3. We applied gene signatures expressed by immune cell populations to early and advanced CAS samples by ssGSEA, a feasible method, and could calculate an order value for each gene based on expression profiles for subsequent immune infiltration analysis. The heterogeneity of immune infiltration among early and advanced CAS plaques and the correlation between different types of immune cells were further explored in GSE43292. We then also performed immune infiltration analysis on the dataset GSE28829 to validate the above analysis results. Moreover, CIBERSORT, a wildly proposed computational algorithm, was used to explore the proportion of 22 immune cell types in early and late CAS. LM22, a leukocyte gene signature matrix containing 547 genes, was used to quantify 22 immune cell types. These 22 immune cells mainly include neutrophils, macrophages, NK cells, plasma cells, B cells, and T cells [43]. We made 1000 permutations and retained samples with p < 0.05 to guarantee the reliability of the results, with the sum of various immune cells being 1.

### Statistical analysis

All data processing, statistical analysis and plotting were carried out using R 4.1.0 software. The statistical significance was compared between the two groups via the student’s *t*-test (for normally distributed data) or the Wilcoxon rank-sum test (for abnormally distributed data). The correlation among variables was evaluated using the Pearson correlation coefficient. The Bonferroni method was applied for adjusting P-value. A two-tailed P-value < 0.05 was considered statistically significant.

## Electronic supplementary material

Below is the link to the electronic supplementary material.

Additional file 1: Supplementary Figures.

Additional file 2: Human autophagy database.

Additional file 3: The forward and reverse primers for qRT-PCR.

Additional file 4: The gene sets for marking 28 immune cell types.

## Data Availability

Public data used in this work can be acquired from the Gene Expression Omnibus (GEO http://www.ncbi.nlm.nih.gov/geo/). The experimental data supporting the conclusions of this article will be made available by the corresponding authors.

## Abbreviations

CAS: carotid atherosclerosis
ATGs: autophagy-related genes
GEO: Gene Expression Omnibus
DE-ATGs: differentially expressed autophagy-related genes
PPI: protein–protein interaction
IHC: Immunohistochemistry
RT-qPCR: Real-time reverse transcription-quantitative polymerase chain reaction
ceRNA: competitive endogenous RNA
AUC: area under the curve
GO: Gene Ontology
KEGG: Kyoto Encyclopedia of Genes and Genomes
ROC: Receiver operating characteristic
ssGSEA: Single-sample gene-set enrichment analysis.
