# Supplementary material for: Comprehensive analysis of autophagy-related gene expression profiles identified five gene biomarkers associated with immune infiltration and advanced plaques in carotid atherosclerosis
Source: Orphanet J Rare Dis. 2023 Mar 23;18:66. doi: 10.1186/s13023-023-02660-2 (PMC10037854; doi:10.1186/s13023-023-02660-2)
Supplement: Supplementary file 1 — Additional file 1: Supplementary Figures. [file 13023_2023_2660_MOESM1_ESM.docx]

**
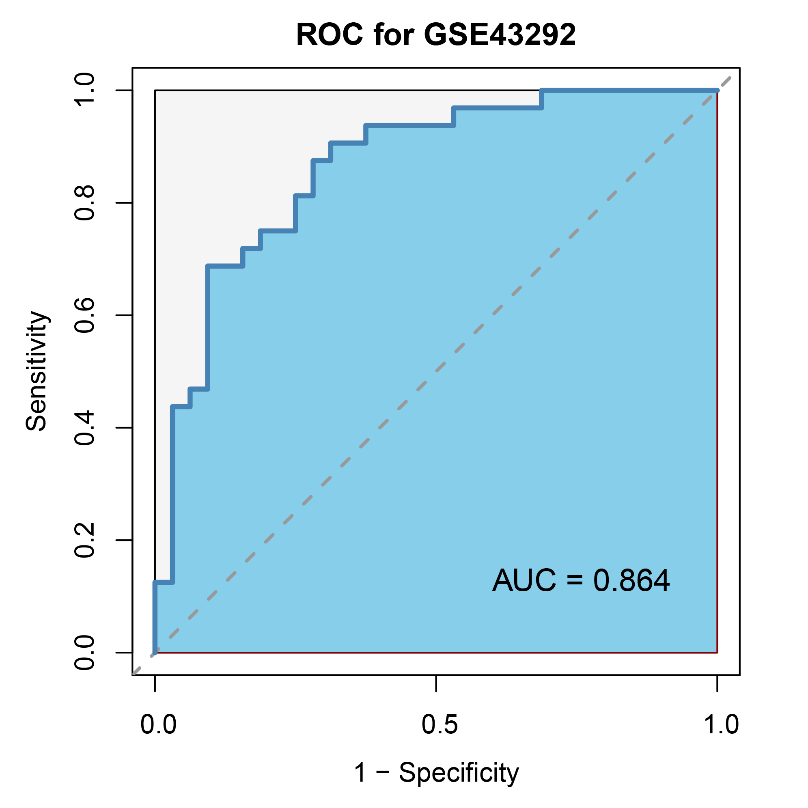
**

**Figure S1. ROC Curve for Logistic Regression Model Combining five hub genes in GSE43292.**

**
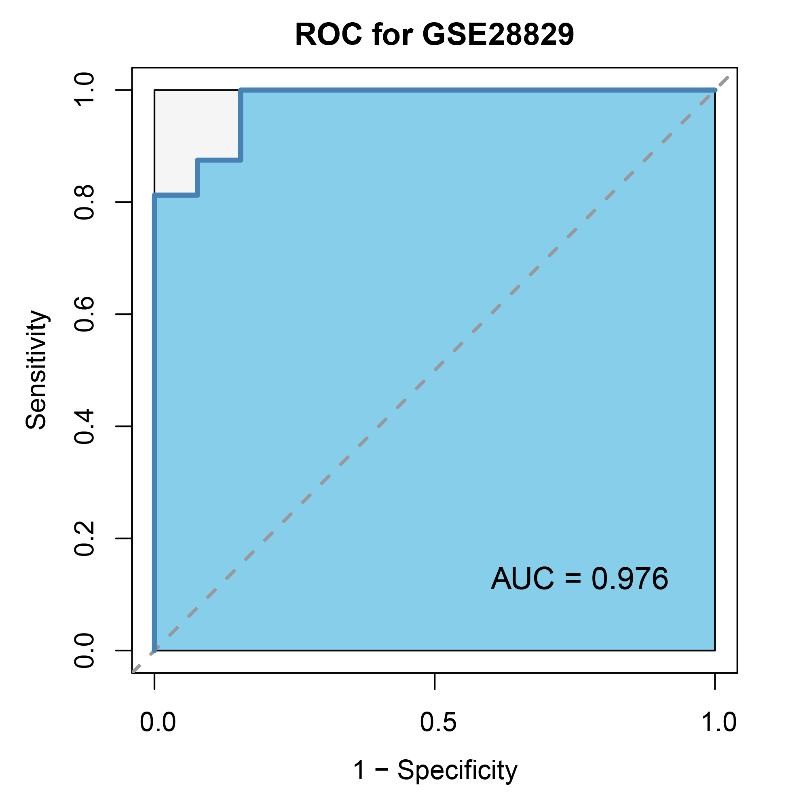
**

**Figure S2. ROC Curve for Logistic Regression Model Combining five hub genes in GSE28829.**

**
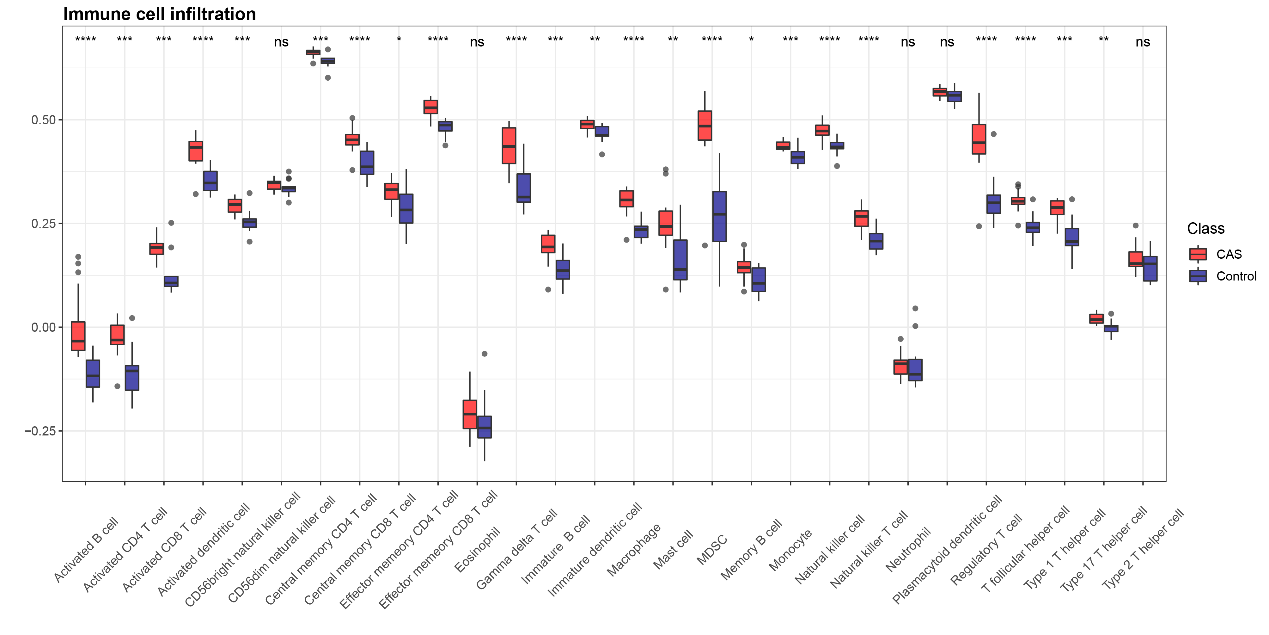
**

**Figure S3. The ssGSEA results were validated in GSE28829.**

**
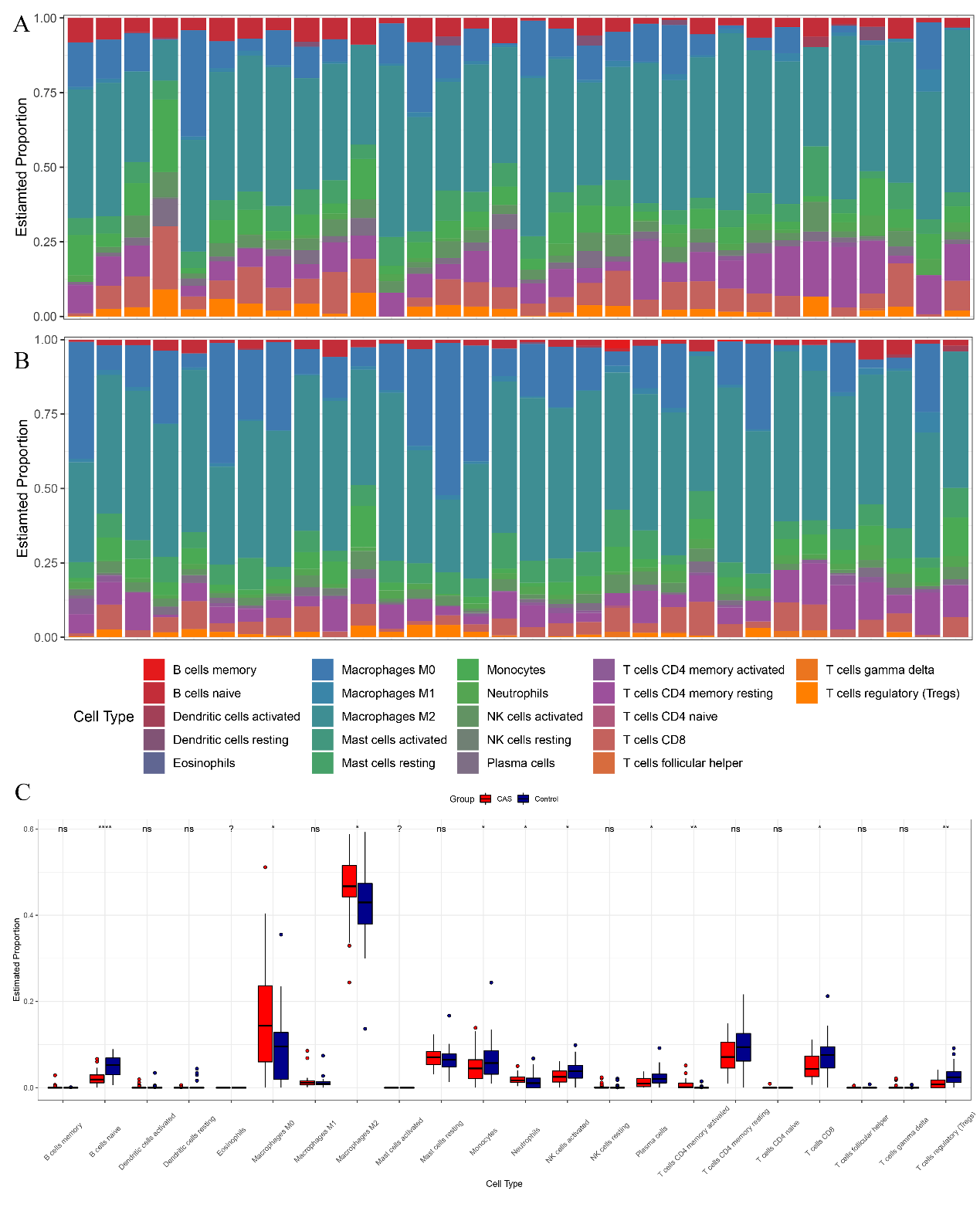
**

**Figure S4. The proportion of 22 immune cells in carotid atherosclerotic plaques. (A) Early plaque. (B) Advanced plaque. (C) Differential analysis of early and advanced plaque.**
